# Self-Sustained Autonomous Wireless Sensor Network with Integrated Solar Photovoltaic System for Internet of Smart Home-Building (IoSHB) Applications

**DOI:** 10.3390/mi12060653

**Published:** 2021-06-02

**Authors:** Md. Rokonuzzaman, Mahmuda Khatun Mishu, Nowshad Amin, Mithulananthan Nadarajah, Rajib Baran Roy, Kazi Sajedur Rahman, Adamu Muhammad Buhari, Shuza Binzaid, Mohammad Shakeri, Jagadeesh Pasupuleti

**Affiliations:** 1 Institute of Sustainable Energy (ISE), Universiti Tenaga Nasional (The National Energy University), Kajang 43000, Selangor, Malaysia; mahmuda.khatun@uniten.edu.my (M.K.M.); mshakeri@uniten.edu.my (M.S.); jagadeesh@uniten.edu.my (J.P.); 2 College of Engineering (COE), Universiti Tenaga Nasional (The National Energy University), Kajang 43000, Selangor, Malaysia; 3 Power and Energy System, School of Information Technology and Electrical Engineering (ITEE), University of Queensland, Brisbane 4072, Australia; mithulan@itee.uq.edu.au; 4 School of Engineering and Technology, Central Queensland University, Bryan Jordan Drive, Gladstone 4680, Australia; rajibbaran.roy@cqumail.com; 5 Solar Energy Research Institute, Universiti Kebangsaan Malaysia (The National University of Malaysia), Bangi 43600, Selangor, Malaysia; sajed@ukm.edu.my; 6 Faculty of Engineering, Multimedia University, Cyberjaya Campus, Cyberjaya 63100, Selangor, Malaysia; adam_m.buhari@yahoo.com; 7 Smart Microgrid Advanced Research and Technology (SMART) Center, Department of Electrical and Computer Engineering, Prairie View A&M University, Prairie View, TX 77446, USA; shbinzaid@pvamu.edu

**Keywords:** solar photovoltaic (PV), internet of smart home-building (IoSHB), energy harvester (EH), low power electronics, internet of things (IoT), wireless sensor network (WSN), autonomous sensors, smart home, smart building

## Abstract

Conventional wireless sensor networks (WSNs) in smart home-building (SHB) are typically driven by batteries, limiting their lifespan and the maximum number of deployable units. To satisfy the energy demand for the next generation of SHB which can interconnect WSNs to make the internet of smart home-building (IoSHB), this study introduces the design and implementation of a 250 mW to 2.3 W energy harvesting device. The proposed device is dynamically autonomous owing to the integration of embedded solar photovoltaic (PV) modules and power storage through a supercapacitor (SC; 5 V, 0.47 F) capable of powering WSNs for 95 s (up to 4.11 V). The deployed device can harvest indoor and outdoor ambient light at a minimum illumination of 50 lux and a maximum illumination of 200 lux. Moreover, the proposed system supports wireless fidelity (Wi-Fi) and Bluetooth Low Energy (BLE) to do data transfer to a webserver as a complete internet of things (IoT) device. A customized android dashboard is further developed for data monitoring on a smartphone. All in all, this self-powered WSN node can interface with the users of the SHBs for displaying ambient data, which demonstrates its promising applicability and stability.

## 1. Introduction

Smart home-building (SHB) equipped with wireless sensor networks (WSNs) have attracted greater public interest in recent times, attributing their ability to improve dwellers comfort cost-competitively. WSN is the network that integrates sensing, computing, and networking to automate data acquisition, analysis, and telemetry. WSNs allow flexible networking and energy consumption in a growing number of applications [1]. In these SHBs, distributed network nodes are composed of low-power electronic devices equipped with sensors and microcontrollers capable of regularly receiving, storing, and transmitting ambient data to a remote host webserver. Many integrated WSN nodes and scalable internet appeals tend the traditional SHB to function as the Internet of Smart Home-Building (IoSHB). Micro-nano power electronic devices are being used on a large scale inside the SHB to increase the comfort of its inhabitants by enhancing convenience, saving electricity, delivering remote surveillance and real-time sensor data monitoring [2,3]. This is implemented using WSN and IoT design, becoming more prevalent in our everyday lives [4,5]. The rapid growth of the IoT in many living environments, such as smart homes, smart offices, and smart buildings, has necessitated the creation of human–machine interfaces (HMIs) [6,7]. Every WSN physical node is fitted with multi-sensor devices for heterogeneous ambient sensing and can also encode, pre-process, send, and receive data to the main controller. In general, intelligent wireless interconnected sensing systems are required to complete all-new IoT technologies [8,9].

In this context, WSN nodes perform the key role in low power consumption with long-distance communication considering the cost, size, and ease of setup and installation [10]. Therefore, the requirement for a wired power source for autonomous wireless devices remains a major concern. The electricity needed for the device is often supplied in the form of removable batteries. Network communications are increasingly evolving from wired to wireless, with all sensors being linked, interoperable, and needing rapid rollout. It is essential to introduce new powering techniques for autonomous sensors, raise technological knowledge, and increase adoption by removing battery transition as a big operational and environmental problem [11]. AmbiMax [12] is a WSN node energy harvesting circuit and SC-based energy storage device proposed to solve the battery ageing problem of the existing WSN energy harvesting system. The SC can charge at the maximum efficiency using the maximum power point tracking (MPPT) technique. Furthermore, AmbiMax is versatile, allowing for the combination of several energy harvesting sources, such as sun, wind, thermal, and vibration, each with a unique optimum scale. Heliomote [13] is a self-contained system operated by a solar panel and two AA nickel–metal hydride (NiMH) batteries. The solar panel is directly attached to the battery through a diode. Furthermore, it lacks MPPT, which is needed for high energy harvesting performance. The authors in [14] discuss the core problems and trade-offs in the design of solar energy harvesting in wireless embedded systems; the design, deployment, and performance assessment of Heliomote. The experimental findings show that Heliomote acts as a plug-in to the Berkeley/Crossbow motes and handles energy harvesting and storage autonomously, allows near-perpetual harvesting conscious activity of the sensor node. The authors in [15] designed a credit card-sized self-powered sensor node to generate 240 µW power and charge the 0.55 F SC in 800 s. Authors [16] designed an ultra-low power of 0.9 mW electromagnetic energy harvester to charge the 12.8 mF SC in 1440 s. Shen et al. presented an electromagnetic device that can generate 21.51–31.6 mW [17]. A 2 mF SC-based piezoelectric energy harvesting WSN is developed in [18] that can generate a maximum of 1.1 mW, with a maximum charging time of 67 s. A 0.47 mF SC can be charged in 125 s and generates 46.06 µW from the vibrations that is presented in [19]. An experimental study of an ultra-low-power WSN energy harvesting system takes 12 h time to charge a 0.33 F SC [20]. An electromagnetic energy harvesting system can generate 11.5 mW and charge the 14.7 mF SC in 30 s that is presented in [21]. An arc-shaped triboelectric nanogenerator (AS-TENG) is designed in [22] to harvest energy from wind and water flow. The AS-TENG achieves an open-circuit voltage of up to 600 V and a short-circuit current of 40 μA, illuminating 248 LEDs instantaneously under a wind speed of 15 m/s. A self-powered piezoelectric sensor-based system with a single electrode is constructed in [23]. This device can detect body motions such as walking, running and the motion of some mechanical devices such as peristaltic pumps, lock, and window switches. The self-powered sensor can warn the user through a smartphone if a door or window is opened by unauthorized personnel.

The authors introduce SeisMote in [24], a modern portable wearable tool for tracking cardiovascular activity. Custom low-power protocols are designed to enable simultaneous control of 32 signals from only 12 nodes at 0.2 ms. The device usage in the field revealed experimental records of data loss, and the battery charge surpassed 16 h. Building automation, agriculture, health and medical, and process monitoring are important sectors where energy harvesting technologies are important. The use of WSN in buildings for better monitoring and maintenance of air handling systems, decreasing building energy use and improving building air quality to ensure occupant well-being is a clear illustration [3,25]. In 2018, residential and non-residential buildings accounted for the largest share of global final energy use of 36%, as well as energy-related carbon dioxide (CO_2_) emissions of 39% [26]. Buildings can achieve smart air quality management by automatically controlling air handling systems based on the real-time CO_2_ concentration, temperature, and humidity, saving up to 25% on energy costs [27]. Since the environmental parameters do not alter often, each sensor node in the IoT needs to function in an active mode for sensing, processing, and communication periodically. The ultralow-power consumption mode has a relatively long-time span, such as in the order of minutes at the power consumption in milliwatt (mW).

In contrast, the power-hungry active mode has a relatively short period, such as millisecond (ms) at the power consumption in mW. Consequently, IoT-based sensor nodes total power usage is much smaller than in active mode. It allows to power an IoT-based sensor node utilizing ambient energy harvesting components, such as PV energy harvesters, where energy collection during the sensor nodes sleep cycle can be continuously accumulated in a battery or supercapacitor [28]. The long lifespan of energy harvesting and storage modules allows the IoT-based sensor node to be driven without thinking about battery replacement. Attempts have been made to combine PV cells, power control circuits, and even storage to create a completely integrated PV energy harvesting system that can eventually contribute to self-powered IoT systems [29]. However, due to the comparatively higher power requirements for wireless data transmission, lowering the IoT nodes overall power usage is challenging. In the case where indoor lighting conditions are dim, which limits the amount of PV energy harvested, the argument for powering an indoor IoT sensor node is more difficult. Therefore, there is insufficient indoor WSN powered by PV energy harvesting mechanism for air quality measurements in buildings. It should also be noted that the majority of studies are just recommendations from software simulation works, with very few being gone for full implementations.

This paper introduces a novel concept of an autonomous low power PV energy integrated WSN framework for innovative home applications to advance both study and performance. A careful hardware and software integration is used to develop a hardware prototype utilizing the existing software platform to achieve long-term sustainable operations. Three software platforms, such as the MATLAB/SIMULINK, simulate the energy harvesting part. The Arduino Integrated Development Environment (IDE) and C++ language are used to program the ESP32 microcontroller, and an android application is used to monitor the system status through a smartphone. As a result of its reduced dimensions and energy autonomy, it is suitable for intelligent home, office, building automation and, more broadly, IoT-enabled sensor applications. WSN nodes provide input for various sensor devices (temperature, humidity, moisture, CO_2_ sensors, etc.) to the consumer or a centralized control host. Low-power activity and energy usage are achieved in careful hardware and software integration in the proposed SHBs. The architecture of the proposed model is presented in Section 2. Section 3 presents the simulation and experimental setup. The simulated and performance test results elaborately discussed in Section 4. This article concludes in Section 5 with highlights of conclusions and contribution of work.

## 2. Architecture of the Proposed Model

**Figure 1:**
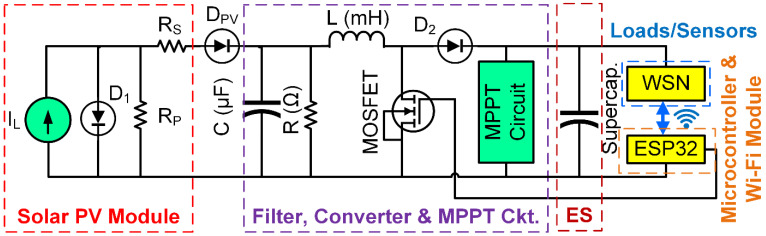
Electrical equivalent circuit of the proposed low-power autonomous solar PV energy harvesting system [5].

A standard solar energy conversion device contains a solar panel, DC-DC converters, rechargeable battery/supercapacitor, battery management unit, low voltage or high voltage protection circuit, and power control algorithm. A mathematical model is developed that adequately describes the solar PV modules non-linear I-V and P-V characteristics. Numerous types show the features of solar cells. To date, the single, double and triple diode is the mostly used equivalent solar circuit model. The single diode equivalent model is used in this article due to its simplicity and precision in describing the complete I-V and P-V curves of a solar cell. A single diode PV module shown in the reference [30,31,32] is used to simulate the proposed model. A buck-boost converter and maximum power point tracking technique (MPPT) is used. Figure 1 shows the electrical equivalent circuit of the proposed energy harvesting model. At the beginning of the circuit, the solar PV module is highlighted with a red dash line followed by the filter circuit, converter circuit, and MPPT block in the purple dash line. The harvested energy from the solar PV module will charge the SC as an energy source (ES) [33]. Currently, to extend the battery life of WSN nodes to months or even years, energy-efficient networking schemes paired with a low-power architecture are used [8,34]. The BLE protocol lowers power usage and enables fast connection to mobile devices. Therefore, it has a narrow operational range and is subjected to royalty, making it unsuitable for pervasive low-cost devices in the WSN [35]. The ESP32 device is chosen as it can work as the microcontroller, Wi-Fi, and BLE module. The SC delivers the required power to the WSN nodes or loads.

**Figure 2:**
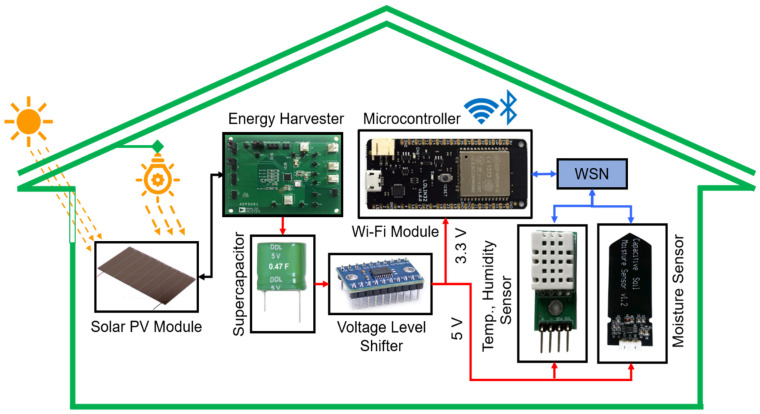
Schematic diagram of the proposed solar PV energy harvesting system for autonomous sensors in smart home applications.

Figure 2 shows the schematic diagram of the proposed solar PV energy harvesting system for autonomous sensors in smart home applications. The solar PV module AM-1816 can work both in the indoor and outdoor environment. Incident light from the direct sun or lamp can operate the solar module. The electrical specifications of the PV module are given in Table 1. This glass type amorphous silicon solar PV module can generate a maximum of 252 µW power, which is insufficient to distribute to the wireless sensor node. Thus, an energy harvesting and power management module is used to increase the power to a certain level to efficiently operate the WSN and the other components in the proposed model. The ADP5091, an intelligent, integrated energy harvesting low power management unit (PMU) that converts DC power from PV cells, is used. These devices charge storage items such as rechargeable lithium-ion batteries, thin-film batteries, supercapacitors, and provide power to small electronic devices and battery-free systems. The output of the solar module is connected to the ADP5091 (Analog Devices Inc., Norwood, MA, USA) energy harvester module. Due to the non-linear characteristics of the solar PV module, the energy storage may suffer an unstable power supply from the source during the charging period. The authors in [36] stated that SCs are suitable when fast charging is needed to meet a short-term power requirement, while batteries are required to provide long-term electric energy. Therefore, a double layer SC is chosen to store the energy for continuous support to the system during the absence of solar power.

The double-layer of the SC ensures fast charging and discharging because it can occur at any moment [37]. Since the SC charges and discharges quickly, a specific condition on the microcontroller is used. Once the SC started to discharge, the sensor data transfer rate will be optimized based on the available charge. If the available energy is 50−100%, the data transfer can happen with a short time interval, whereas the time interval will be increased if the charge level is reduced below 50%. In this way, the SC can back up the system uninterruptedly for a long time. Moreover, as the shift in the atmosphere inside the SHB is gradual, the sensor device does not require to transmit signals continuously. Instead, the device will be in standby mode during the usable solar PV electricity, which is continuously collected, and then turn on for sensing and signal to transmit for a short period. Such a periodic function of the sensor device is feasible for creating a self-powered WSN with long-term operation. A voltage level shifter is associated with the SC and can distribute the voltage of 3.3 V to the Wi-Fi module ESP32 and 5 V to the sensors. Sensors are connected with the Wi-Fi module through the WSN node.

## 3. Simulation and Experimental Setup

Both the simulation and experimental tests have been carried out to validate the proposed model. Except for few differences in some points, both the simulation and experimental setup shows the desired results. The simulation is carried out in MATLAB/SIMULINK 2020b (MathWorks, Natick, MA, USA) environment.

**Figure 3:**
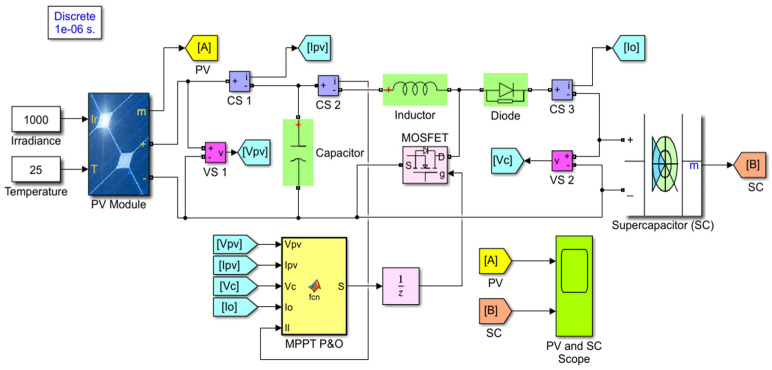
Proposed simulation model of the solar PV energy harvesting model.

**Figure 4:**
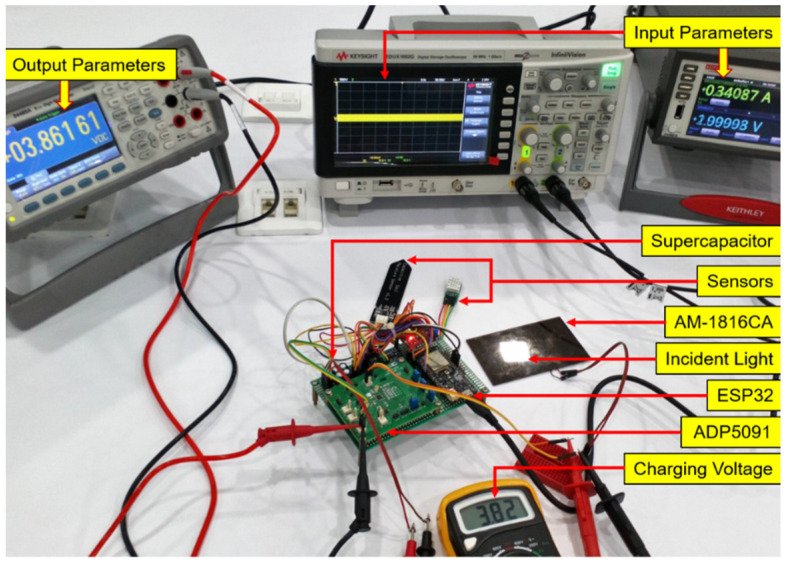
Experimental test bench in the laboratory, including energy source as fluorescent light, AM-1816 PV module, prototype device, wireless sensor nodes, and relevant measurement devices.

The proposed simulation model is shown as the SIMULINK block in Figure 3. In the simulation, irradiance takes a minimum of 200 W/m^2^ to a maximum of 1000 W/m^2^ with a 200 W/m^2^ interval. The temperature is taken a minimum of 15 °C to a maximum of 55 °C. A perturb and observe (P&O) algorithm extracts the power from the solar PV module at the MPP [39]. The complete experimental setup is shown in Figure 4. The input parameters, PV voltage, current, and power, are observed by the source meter (model no. 2460, KEITHLEY, Cleveland, Ohio, USA). Input voltages are monitored by the digital storage oscilloscope (model no. EDUX1002G, Keysight, Santa Rosa, CA, USA). Output voltage and current of the PV energy harvesting device is measured and visualized by the digit multimeter (model no. 34465A, Keysight, Santa Rosa, CA, USA). The digital multimeter measures supercapacitor charging voltage. The AM-1816CA (Panasonic Electric Works, Ottobrunn, Germany) amorphous silicon solar module is used as the primary energy source. The fluorescent (FL) incident light from the ceiling is captured on the solar module.

## 4. Results and Discussions

**Figure 5:**
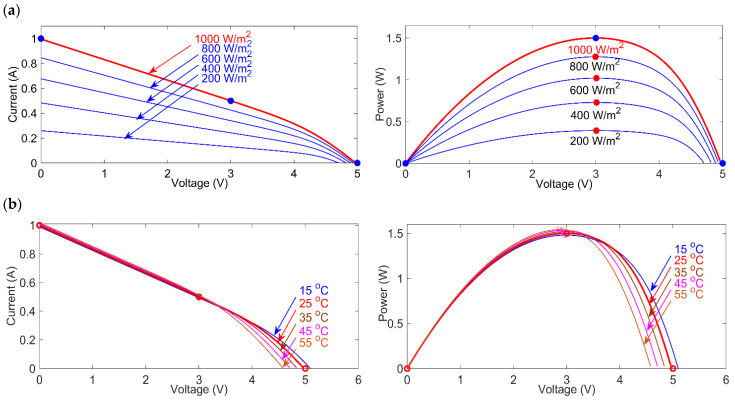
Solar module AM-1816 characteristics (**a**) I-V and P-V characteristics at different irradiance (W/m^2^), (**b**) I-V and P-V characteristics at different temperature (°C).

A complete prototype of a solar PV energy harvesting (EH) system is developed and validated in a smart home environment. The designed EH device and IoT system are capable of operating three IoT-connected sensors. Following the implementation of the configuration of the hardware, the model goes through a simulation. The simulation predicts the optimal outcome. Figure 5 shows the different characteristics curve of the AM-1816 solar PV module. The performance difference of a solar PV cell highly depends on the irradiance and temperature [40,41]. The solar cell shows the highest I-V at the condition of the highest irradiance and lowest temperature. At the same time, the lowest I-V output is generated at the lowest irradiance and highest temperature. In terms of the effect of irradiance on the properties of a PV module, the induced current is proportional to the total incident irradiance with a dependency at constant temperature and can be expressed by Equation (1). The short circuit current at 25 °C is Isc, α is the short circuit current correction coefficient, the solar cell operating temperature is *Tc*, and the incident irradiance $I_{SC}\;\left(T\right)$ G.

(1) $$I_{SC}\;\left(T\right)=I_{SC}\;\left(at\;25\;℃\right)\;\left[1+\alpha \left(T_{C}-25\right)\right]\;\frac{G}{1000\;W/m^{2}\;}$$

The left side of Figure 5a shows the I-V curve at different irradiance 200 W/m^2^ to 100 W/m^2^. The maximum current of 1 A and maximum voltage of 5 V is generated at the maximum irradiance of 1000 W/m^2^, whereas the minimum current and voltage are generated at the minimum irradiance of 200 W/m^2^. Similarly, the right side of Figure 5a shows the P-V curve. The highest power is generated at the highest irradiance at 1000 W/m^2^. When all other factors remain unchanged, the higher the temperature, the lower the open-circuit voltage of the solar PV cell. This is referred to as a power loss. On the other side, when the temperature falls compared to the initial conditions, the PV output voltage and power increases. The voltage dependency on temperature is expressed in [40] as a function of $V_{OC}\;\left(T\right)$ standard temperature condition (STC).

(2) $$V_{OC}\;\left(T\right)=V_{OC}\;\left(at\;25\;℃\right)\;\left[1+\beta \left(T_{C}-25\right)\right]$$

Here, the open-circuit voltage temperature coefficient is β, and the solar cell operating temperature is $T_{C}$. As the solar cell temperature rises, the semiconductor bandgap narrows, allowing more energy to be consumed, increasing the solar cells short circuit current for a given irradiance. Simultaneously, increasing the temperature rapidly expands the population of electrons. The simulation of the I-V and P-V characteristics of the solar PV module under different temperatures is carried out for indoor and outdoor applications. The left side of Figure 5b shows the maximum voltage is generated at 25 °C comparing to the minimum temperature of 15 °C and maximum temperature of 55 °C. The right side of Figure 5b shows the P-V curve at different temperature readings.

**Figure 6:**
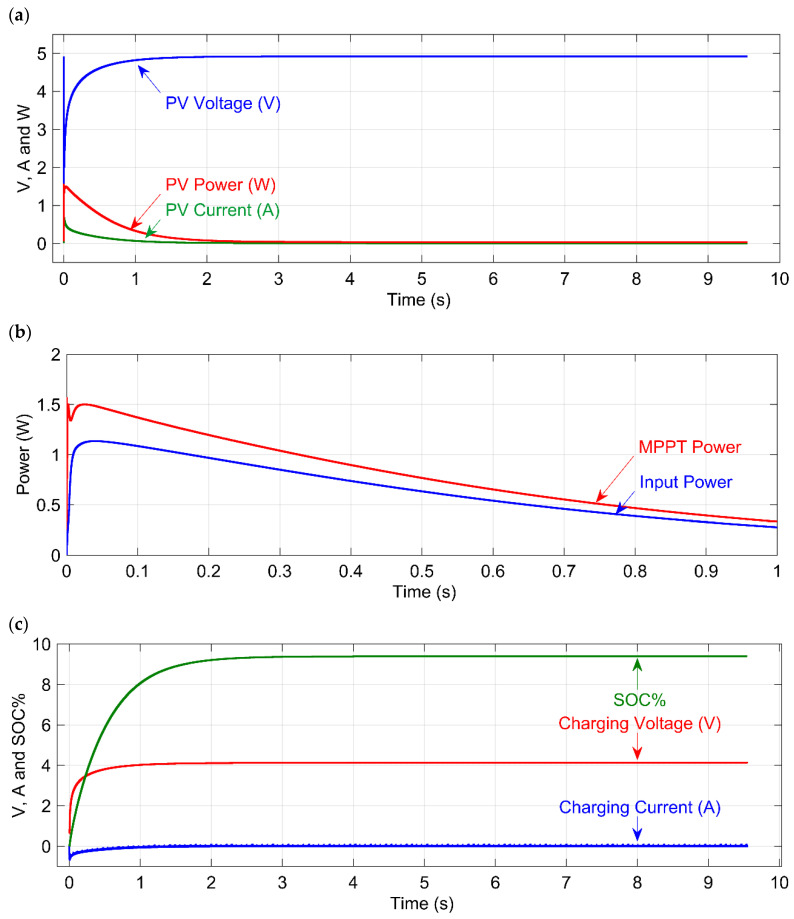
Simulation results; (**a**) generated voltage (V), current (A), and power (W) from the solar PV module; (**b**) generated power (W) without MPPT tracker and power with MPPT tracker (W); (**c**) charging voltage (V), charging current (A), and SOC (%) of the SC.

Figure 6 depicts the proposed models simulated performance. As shown in Figure 6a, the induced voltage increases to a maximum of 4.8 V after 2 s. Whereas the systems peak power was initially 1.2 W, it now offers steady power of about 0.5 W. The power of without and with MPPT control is depicted in Figure 6b. The proposed model achieves a maximum MPPT power of 1.5 W, compared to 1.2 W without the MPPT tracker. Both MPPT and normal power decreases as time passed. The charging voltage, charging current, and state of charge (SOC%) characteristics of the SC are shown in Figure 6c. The SCs state of charge is 9.39% after 9.5 s; the optimal performance voltage level is 3.3 V, which can be completed in less than 0.1 s. Later, in the absence of energy sources, the SC maintains a steady voltage of 3.3 V. The simulation demonstrates that charging the SC to 4 V takes just 1 s. Only the energy sources and energy harvester components are simulated and evaluated in this analysis. The solar cell is attached to the Vin of the ADP5091 energy harvester module in the prototype. Vin is connected internally to the charge pumps cold start and the MPPT pin. The performance of the MPPT pin is attached to the boost controller, with the boosted voltage being delivered to the modules BAT pin. The BAT pin is coupled directly to the SC. The experimental results show the SC begins to charge when the energy supply produces just 0.8 V. The experiments evaluate various input voltage levels ranging from 0.8 V to 3.2 V.

**Figure 7:**
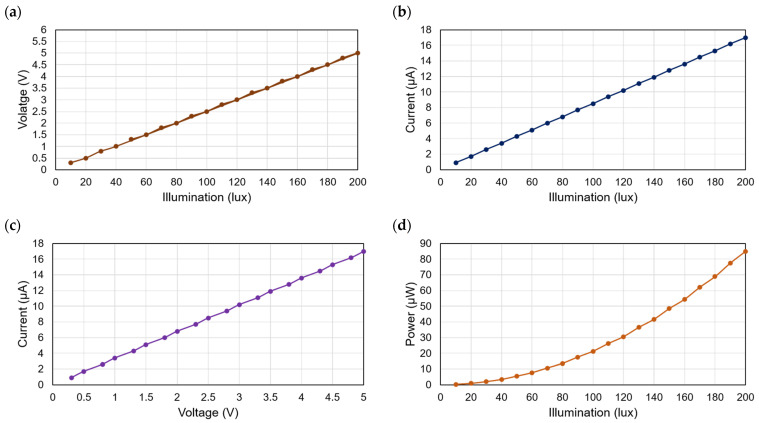
Different characteristics of the AM-1816 PV module; (**a**) illumination vs. voltage; (**b**) illumination vs. current; (**c**) current vs. voltage (I-V); and (**d**) illumination vs. power.

Figure 7 illustrates the different features of the PV module as illuminated by various light sources. The variation of illumination vs. voltage of the PV module is depicted in Figure 7a. Each cell of the AM-1816 solar PV module produces a voltage of 0.63 V at the illumination of 200 lux in an indoor environment. A voltage of 0.89 V at an irradiance of 1000 W/m^2^ in the outdoor environment. The PV modules illumination vs. current characteristics is depicted in Figure 7b. The module produces 4.3 µA current at a minimum of 50 lux and a 17 µA current at a maximum illumination of 200 lux. The I-V characteristics of the AM-1816 module are shown in Figure 7c, accompanied by the illumination vs. power graph in Figure 7d. The module can deliver 85 µW power at an optimum illumination level of 200 lux.

**Figure 8:**
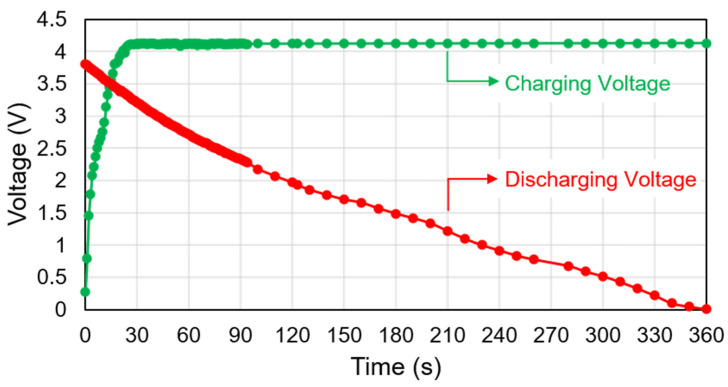
Charging and discharging state of the supercapacitor.

Figure 8 depicts the SC in its complete charging and discharging mode. The SC is found to cross 4.12 V in less than 30 s. At a maximum generated PV voltage of 3.2 V at 130 lux, it is observed that the proposed device needs just 17 s to charge. Charging the SC at the lowest voltage standard of 0.8 V (at 30 lux) takes a time of 185 s. For calculation purposes, the SCs maximum charging voltage level has been set to 3.82 V. It takes 118 s, 62 s, 42 s, 27 s, and 18 s for the proposed system to operate at various input voltages of 1 V, 1.5 V, 2 V, 2.5 V, and 3 V, respectively. Thus, the low input voltage requires more time to charge from the experiment. In comparison, the high input voltage requires less time to charge. Additionally, it is observed that the SC charges to 4.11 V in 25 s. The voltage level is increased to 4.12 V after 30 s. Throughout the measurement procedure, a steady SC voltage of 3.82 is used and the SC discharges from fully charged voltage of 3.82 V to 0 V. The estimated discharge time is 95 s to discharge the SC from 3.82 V to 2.28 V. As a result, the sensors and IoT feature run continuously for 95 s without external energy sources. It is also observed that the SC requires approximately 360 s to discharge to 0 V during the circuit finally is on mode.

For the charging state, the charging time depends on the illumination of light and the voltage generated from the PV module. For discharging, the SC discharges very fast during the data transmission to the web browser and smartphone. The wireless sensor node operates at a minimum voltage of 1.97 V for the moisture sensor. For the temperature and humidity sensor, at least 2.28 V is required. Until the SC voltage reaches 2.28 V, the sensors transmit data to the IoT server. The temperature sensor (SHTC3) and moisture sensor (SEN0193) cannot connect to the server when the charge amount is less than 2.28 V. The used SC discharges from 3.82 V to 1 V in 230 s. It is found that the moisture sensor starts transmitting the data at a voltage of 1.97 V. The SHTC3 sensor continues to run, but the power supply is inadequate to relay the data. SHTC3 transmits data to the internet server through the ESP32 Wi-Fi module at a voltage of 2.28 V. Thus, during the discharge, the SC powers up the SHTC3 and SEN0193 sensors for 95 s (at a minimum of 2.28 V) and 120 s (at a minimum of 1.97 V), respectively.

**Figure 9:**
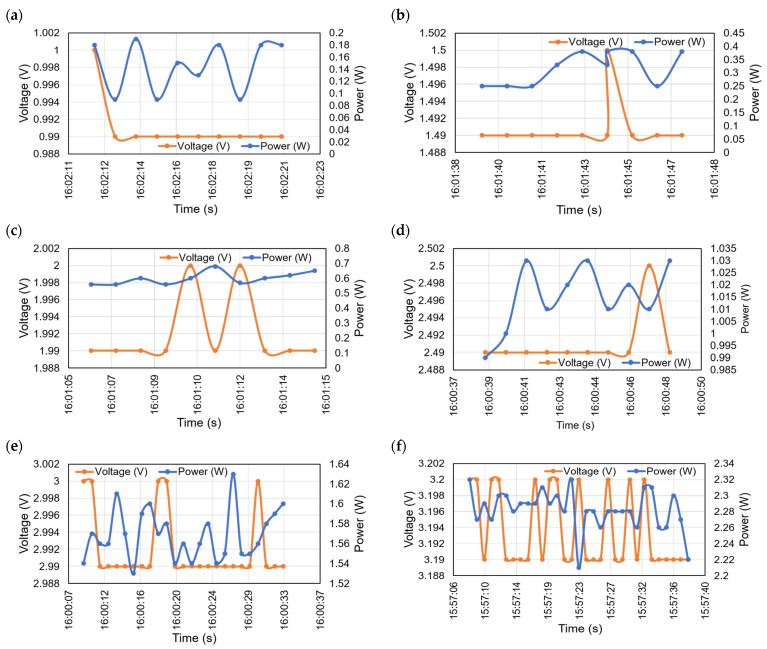
Generated voltage and power from the proposed system at different illumination level and time; (**a**) voltage and power vs. time at 40 lux; (**b**) voltage and power vs. time at 60 lux; (**c**) voltage and power vs. time at 80 lux; (**d**) voltage and power vs. time at 100 lux; (**e**) voltage and power vs. time at 120 lux; (**f**) voltage and power vs. time at 130 lux.

Figure 9 shows the experimental voltage and power waveforms generated at a different time with different illumination levels. Figure 9a shows the generated power and voltages at the illumination of 40 lux. The highest and the lowest peak power at this stage is 0.19 W and 0.09 W, respectively, whereas the generated voltage is around 1 V. Figure 9b shows the generated power at the highest peak power of 0.38 W and the lowest peak power of 0.25 W at the illumination of 60 lux and voltage of around 1.5 V. Figure 9c shows the highest peak power of 0.68 W and the lowest peak power of 0.56 W at the 80 lux and the generated voltage is around of 2 V. Figure 9d shows the highest peak power of 1.03 W at the 100 lux and the generated voltage is 2.5 V. Figure 9e shows the maximum generated power is 1.63 W at 120 lux and around 3 V. Figure 9f shows the highest peak power of the proposed system is 2.3 W at the highest illumination level of 130 lux and around voltage of 3.2 V. Since the system is designed for low power and indoor environment, here the maximum illumination of 130 lux is taken in account. Therefore, the proposed approach can generate the highest voltage of 5 V with the highest illumination level of 200 lux.

Table 2 displays the details on the power usage of the wireless sensor. 1.885 × 10^−5^ W is absorbed by the temperature and humidity sensor. The moisture sensor absorbs 0.020304 W power. The sensors standby state and data transfer mode use a total of 0.20011885 W. The proposed systems generated power (P_GEN_) is 0.25 W, which is enough to operate the wireless sensor node at the minimum illumination of 50 lux. The experimental results show that when data is transmitted at two seconds, the SC can back up the whole device for 95 s. At this stage, sensors successfully transfer 40 data strings to the webserver. A higher or lower sensor data sampling rate may result in increased or decreased power consumption, respectively. Thus, depending on the devices usable resources or state of charge, it is also possible to save SC power by adjusting the data transmission interval and data sampling rate. Appropriate resource optimization based on the data transmission rate and the sleep/wake-up time of the sensors will result in significant power savings. The capacitive moisture sensor is a power-hungry device comparatively the temperature and humidity sensor from the experiments. Thus, the authors suggest using an alternative capacitive moisture sensor for commercial and efficient low power consumption.

**Figure 10:**
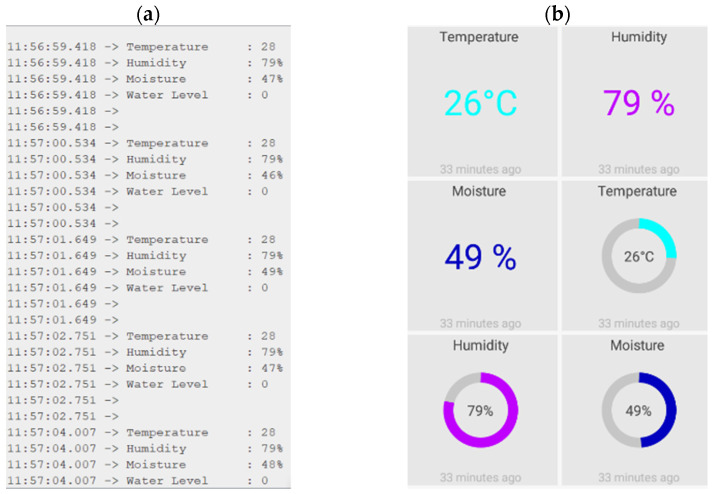
IoT features of the proposed prototype, (**a**) transmitting data to the webserver; (**b**) customized android dashboard on the smartphone.

The developed IoT architecture is fully operational, both in the webserver and through an android application. Figure 10a,b shows the data transmission to the webserver and data monitoring customized dashboard on the smartphone through the android application. If the system detects an active Wi-Fi network, it will connect to it; otherwise, it will enable Bluetooth mode and attach to the handset. Figure 10a shows the proposed prototype connected to the server, with the captured data shown on the laptop serial monitor. Figure 10b depicts the live message queuing telemetry transport (MQTT) dashboard, which constantly updates the sensor data inside the smart home. The MQTT software is available on the google play store and can be used free of cost. This proposed application is customized based on the requirement and the number of data slot displaying on the smartphone screen is selected based on the available sensors. The output parameters of the temperature, humidity, and moisture sensors are shown on the dashboard in Figure 10b.

Recently published energy harvesting sensor platforms are presented in the literature. The authors compare the proposed design with the existing work on solar integrated energy harvesting systems for WSNs in Table 3 [42]. The authors in [43] present an intelligent MPPT solar energy harvesting (SEH) system for ZigBee based WSNs that can harvest 450 mW power. The generated power is stored in lithium (Li) battery. The MPPT SEH system is designed for Crossbow motes based wireless embedded systems in [14] that stores the energy to the nickel–metal hydride battery (NiMH). A solar energy harvester is proposed in [44] for WirelessHART based industrial wireless sensor nodes, the generated energy stores in a Lithium polymer (LiPO) battery. The authors in [45] propose a micro solar MPPT power sensor network, Tmote Sky WSN motes and NiMH, as the wireless sensor applications and energy storage, respectively. The SEH circuit is developed for Tmote Sky based WSNs embedded systems [46]. Crossbow Mica2 WSNs is used by solar and NiMH battery-powered energy harvesting systems in [47]. An indoor solar and ultracapacitor (UC) based energy harvesting system is proposed for Crossbow MicaZ sensor network router nodes [48]. Hua Yu et al. [49] proposed an indoor light energy harvesting system for temperature (Temp.) and humidity (Hum.) sensor-based energy-aware wireless sensor node. The proposed prototype generates a maximum of 2.3 W power utilizing the MPPT techniques. An SC of 0.47 F is used to store the energy, which takes only 25 s time to charge fully. The ESP32 module can perform both as the Wi-Fi and Bluetooth low energy (BLE) mode to save energy. The wireless sensor node includes temperature, humidity, and moisture sensors. Compared to other existing work mentioned in Table 3, the proposed system can generate the required power to operate the wireless sensor network that includes the maximum number of sensors and takes less time to charge the SC fully. Wi-Fi and BLE allow to perform the device as self-sustained based on the available power.

## 5. Conclusions

This paper proposes an autonomous energy harvesting system with a low power solar PV module for indoor and outdoor usages for smart home-building (SHB) applications. The developed device is entirely functional during an accidental power outage or even in the absence of regular grid electricity. The sensor data can be monitored through the IoT on a smartphone dashboard and anywhere through an internet webserver. Particularly, three sensors, such as temperature, humidity, and moisture, are tested, and the total consumed power by the sensors is 200 mW (0.20011885 W). In contrast, the proposed energy harvesting system can generate 250 mW (0.25 W) at the minimum illumination of 50 lux under any indoor ambience (fluorescent or LED lamp). The proposed device can generate a maximum of 2.3 W power at the maximum illumination of 130 lux. The SC as energy storage enables fast charging up to 4.11 V within 25 s. Once the SC is fully charged, it can back up the complete WSN nodes for 95 s uninterruptedly and transfer a total of 40 data string per 2 s interval to the webserver. The developed self-powered energy harvesting system can power billions of sensors autonomously, especially the sensors used in smart home-building applications.

## Figures and Tables

**Table 1:** Electrical specifications of the AM-1816 Solar PV module [38].

Parameter	Value
Model Number	AM-1816
Substrate	Glass
Type	Amorphous Silicon
Maximum Output Power	252 µW
Open Circuit Voltage, V_OC_	5 V
Short Circuit Current, I_SC_	96.7 µA
No. of Cells	8
Operating Voltage, V_ope_	3 V
Operating Current, I_ope_	92.2 µA
Dimension (W × L × T)	96.7 × 56.7 × 1.1
Weight	15.6 g

**Table 2:** Measurements of the generated power (P_GEN_) and consumed power.

Sensor Name	Voltage	Current	Power	Total Consumed Power	P_GEN_ at 50 lux	P_GEN_ at 130 lux
Temperature and Humidity	3.77 V	0.005 mA	1.885 × 10^−5^ W	0.20011885 W	0.25 W	2.3 W
Moisture	3.76 V	5.4 mA	0.020304 W			

**Table 3:** Comparative analysis with published literature on solar integrated energy harvesting systems for wireless sensor networks vs. the proposed work.

References	EHT	WSN D/A	ES	Available P/E	Env.
Yin Li et al. [43]	MPPT	ZigBee	Li	5.0 V, 450 mW	OD
Vijay R. et al. [14]	MPPT	Crossbow motes	NiMH	4.0 V, 100 mA	OD
R. Ibrahim et al. [44]	-	WirelessHART	Li-PO	21.5 V, 520 mA	OD
Jay Taneja et al. [45]	MPPT	Tmote Sky	NiMH	4.23 V, 111.2 mA	OD
D. Brunelli et al. [46]	-	Tmote Sky	SC	50 mW	OD
P. Corke et al. [47]	MPPT	Crossbow Mica2	NiMH	4 V, 300 mA	OD
A. Hande et al. [48]	-	Crossbow MicaZ	UC	3.24 V, 25 mA	ID
Hua Yu et al. [49]	MPPT	Hum. and Temp. Sensor	SC	4.5 V/72.74 μW	ID
Proposed Work	MPTT	ESP32, Temp., Hum., and Moisture Sensor	SC	5 V, 250 mW–2.3 W	OD and ID

EHT—Energy harvesting technique, D/A—Device/Applications, ES—Energy Storage, P/E—Power/Energy, Env.—Environment, OD—Outdoor, ID—Indoor.

## Data Availability

Not applicable.
